# Case Report: Apical periodontitis due to calculus-like deposit on the external surface of the root apex

**DOI:** 10.3389/froh.2025.1615050

**Published:** 2025-06-12

**Authors:** Esther Muñoz-Soto, Firas Elmsmari, Okba Mahmoud, José Antonio González

**Affiliations:** ^1^ Department of Stomatology, Faculty of Dentistry, University of Granada, Colegio Máximo de Cartuja, Granada, Spain; ^2^ Department of Clinical Sciences, College of Dentistry, Ajman University, Ajman, United Arab Emirates; ^3^ Centre of Medical and Bio-allied Health Sciences Research, Ajman University, Ajman, United Arab Emirates; ^4^ Department of Endodontics, Faculty of Dentistry, Universitat Internacional de Catalunya, Barcelona, Spain

**Keywords:** intentional replantation, nonsurgical root canal retreatment, tooth calculus, apical periodontitis, persistent periapical lesion

## Abstract

**Purpose:**

Herein, we report a rare case of chronic apical periodontitis associated with an extraradicular calculus-like deposit on the root apex of a mandibular left central incisor that was previously treated with root canal therapy.

**Case presentation:**

A 42-year-old man presented with persistent sinus tract formation. Despite non-surgical retreatment, the symptoms persisted, and radiographic evaluations, including cone-beam computed tomography, revealed a periapical radiolucency with radiopaque convexities in the apical third of the root. Intentional replantation (IR) was performed to allow direct clinical access for diagnosis and management. Upon extraction, a dark brown, calculus-like deposit firmly attached to the external root surface was observed. After deposit removal, root-end resection and retrograde filling were performed before replantation. Follow-ups at 3 months and 1 year revealed complete healing of the sinus tract and significant radiographic improvements. This case highlights the role of extraradicular biofilms and apical mineralized deposits in persistent periapical inflammation. Sinus tracts may facilitate mineral-ion migration and contribute to the formation of extra-radicular calculi. Mineralized biofilms may not be resolved using orthograde approaches, necessitating surgical intervention.

**Conclusion:**

IR enables thorough inspection and removal of radicular deposits, offering a minimally invasive and successful alternative to conventional apical surgery. The findings in this case are consistent with those in previous studies suggesting the usefulness of IR for managing refractory periapical lesions caused by extraradicular infections or mineralized biofilms.

## 1 Introduction

The root canal system and supporting structures influence each other during health, function, and disease. Microbial colonization of the root canal system is the main etiological factor in the development and maintenance of apical periodontitis (1). Pulp infection, traumatic injury, and failure of endodontic treatment are frequent causes of this disease (2).

Apical periodontitis is a destructive inflammatory condition that occurs because of the spread of infection from the root canal system to the periapical area through the apical foramina. *In vivo*, this process promotes immune responses that result in inflammatory-cell infiltration, periapical tissue destruction, and hard-tissue resorption (3). On radiographic examinations, these changes typically appear as radiolucent areas (4).

Chronic apical periodontitis is induced by persistent periapical infection and pathogenic stimuli. It is characterized by breakdown of the alveolar bone and production of inflammatory granulation tissues. Chronic apical periodontitis may present as periapical granulomas, chronic periapical abscesses, periapical cysts, periapical condensing osteitis, or sinus tracts (5). Endodontic treatment, which aims to diminish the effect of pathogens in the root canal system on the periapical tissues and eradicate the cause of persistent inflammation, is a useful method to preserve diseased teeth (6).

The persistence or reintroduction of intraradicular microorganisms, extraradicular infections, foreign body reactions, or true cysts represents some of the etiologies contributing to persistent apical periodontitis. The failure of endodontic treatment has been associated with the presence of mineralized bacterial biofilms or apical calculus on the external root surface in cases of extraradicular infections. The initial documentation regarding the existence of calculus-like material covering the root apices was provided by authors such as Rud and Andreasen in 1972 (7). Subsequent research has further substantiated the presence of calcified deposits on the external root surface, which are frequently associated with sinus tracts (1, 8, 9). By employing techniques such as microscopy, the composition of these calculus-like deposits has been systematically assessed.

Extraradicular bacterial biofilms are inherently associated with apical periodontitis, as they enable microbes to survive and maintain infection outside of the root canal system. Numerous studies have documented their association with treatment failure; however, the formation of these biofilms is considered to be infrequent in cases of apical periodontitis, wherein Ricucci and Siqueira observed incidences of approximately 6% (10). In a study conducted by Song et al. in 2011 (11), which analyzed the causes of nonsurgical treatment failure through microscopic inspection during endodontic microsurgery, apical calculus was identified as a potential cause in 1.8% of the 493 teeth surgically evaluated. A recent systematic review of clinical cases by Pérez et al. (12) highlighted the presence of calculus-like deposits in six of the fifteen reported cases of extraradicular infection. Although apical calculus and extraradicular biofilms are rare or challenging to detect via routine clinical or radiographic examinations, these clinical and microscopic findings substantiate that they can be legitimate causes of endodontic treatment failure, often necessitating surgical intervention to rectify persistent apical periodontitis.

Dental calculus is a mineralized bacterial plaque that accumulates on the surfaces of teeth, dental prostheses and restorations (13). Some microorganisms can survive outside the root canal and cause periapical inflammation (14). Several studies have shown that these microorganisms can form an extra-radicular biofilm that adheres to the cementum of the tooth around the root apex and forms small calculus-like mineral deposits (8, 10), and endodontic treatment failure due to the presence of bacteria on the root surface of the tooth has been reported (15). Although the exact mechanism of their formation remains hypothetical, it is believed to involve the mineralization of a bacterial biofilm on the external root surface. Mineral ions could originate from the inflammatory exudate of the apical lesion or from oral fluids migrating through the sinus tract, which is suggested to facilitate this process (16).

Herein, we report a case of chronic apical periodontitis associated with radiopaque areas in the periapical region of the mandibular left central incisor in a 42-year-old man who presented with repeated inflammation and a persistent sinus tract with pus discharge associated with the left mandibular central incisor. The patient's physical status was classified as American Society of Anesthesiology 1, and he had undergone root canal treatment (RCT) in the bilateral mandibular central incisors at another clinic 10 years prior owing to subluxation and color change due to trauma.

**Figure 1:**
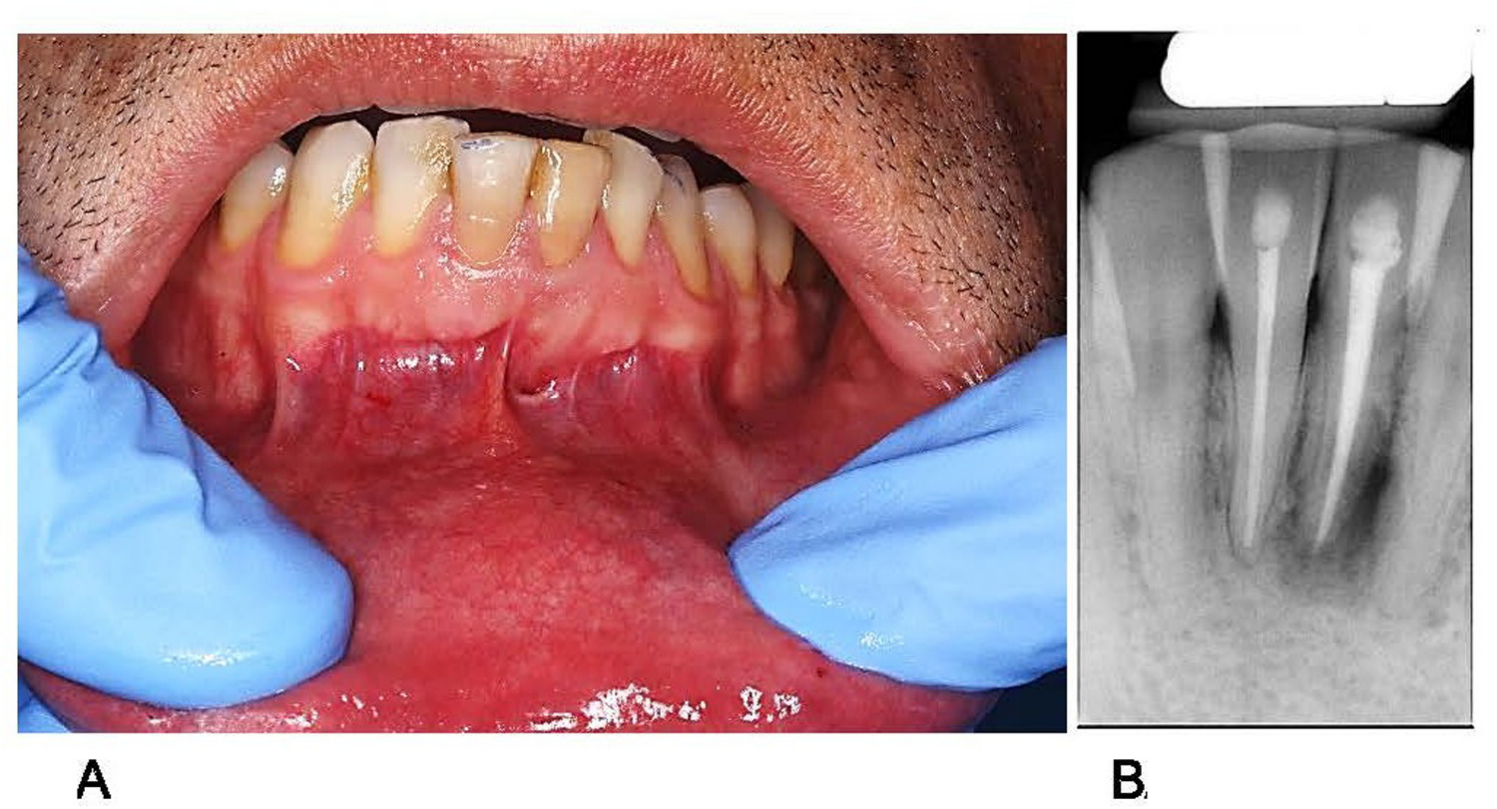
Preoperative condition of tooth #31. **(A)** Clinical photograph **(B)** periapical radiograph.

Clinical examination revealed dental restorations on the lingual surfaces of the mandibular central incisors; however, no developmental abnormalities were noted. Tooth #31 was slightly darker than the adjacent teeth (Figure 1A), and percussion test results were negative. Periodontal examination revealed a sinus tract in the labial mucosa, through which pus was expressed upon pressing. The probing depths and degree of tooth movement were within the normal limits. Periapical radiography (Figure 1B) revealed optimal-quality root canal fillings with in the bilateral mandibular central incisors. However, the mandibular left central incisor showed slight apical root resorption with a large periradicular radiolucent area extending from the mesial aspect of the central incisor to the distal radicular aspect of the left lateral incisor. Based on the clinical and radiographic findings, the mandibular left central incisor was diagnosed as a endodontically treated teeth with chronic apical periodontitis and a buccal sinus tract.

## 2 Clinical procedures

**Figure 2:**
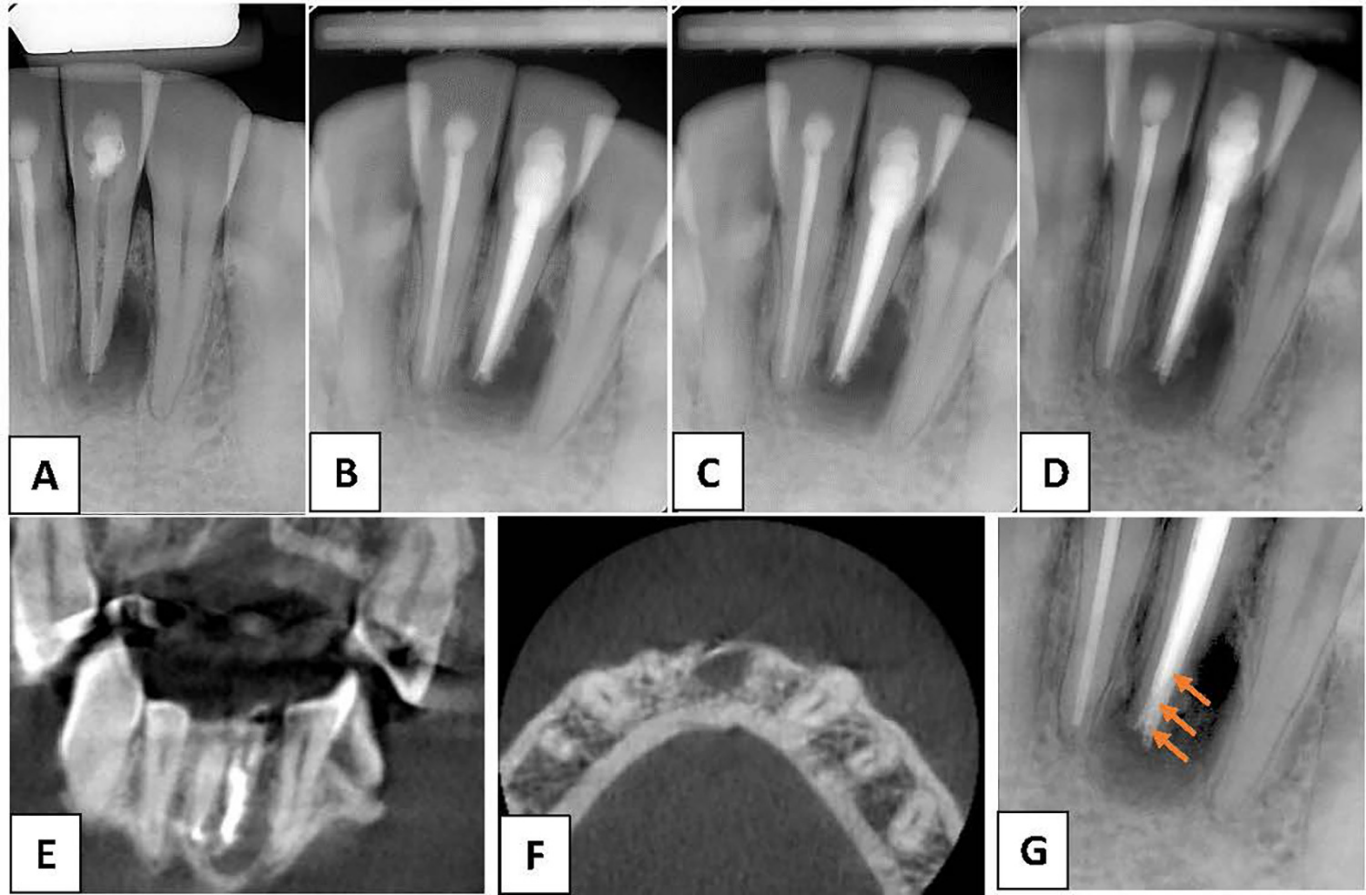
**(A)** Periapical radiograph after the start of nonsurgical root canal retreatment confirms removal of the root canal filling material. **(B)** Periapical radiograph after completion of the nonsurgical root canal retreatment. **(C)** Periapical radiograph at the 6-month follow-up. **(D)** Periapical radiograph at the 2-year follow-up. **(E,F)** Cone-beam computed tomography images at the 2-year follow-up. **(G)** Periapical radiograph at ___ shows radiopaque convexities (arrows) on distal aspect of the external root surface of tooth #31 associated with a radiolucent area.

After obtaining informed consent from the patient, nonsurgical endodontic retreatment of tooth #31 was performed in multiple visits. The patient's medical status was classified as ASAI (American Society of Anaesthesiologists classification). The access cavity was reopened under block anesthesia using articaine 40 mg/ml + epinephrine 10 µl/ml (Artinibsa, Inibsa, Barcelona, Spain). The filling material was removed using Reciproc R25® files (VDW, Munich, Germany), and the canal was prepared up to size 45/.04 using Profile® files (Dentsply Sirona, Bellaigues Switzerland) with copious irrigation using a 5.25% NaOCl solution (Figure 2A). The canal was dried and dressed using pure calcium hydroxide paste, and the access cavity was sealed using a temporary restoration. After 14 days, the temporary restoration and calcium hydroxide paste were removed and the root canal was irrigated with 5.25% NaOCl and 10% citric acid. The canal was dried and filled with Thermafil (Dentsply Sirona, Bellaigues Switzerland) and AH Plus sealer (Dentsply Sirona, Bellaigues Switzerland). Postoperative radiography demonstrated optimal-quality root canal obturation with no sealer or gutta-percha extrusion into the periradicular tissue (Figure 2B).

At the 6-month recall, periapical radiography revealed no change in the periapical radiolucency around the mandibular left central incisor (Figure 2C) compared to that on the previous radiograph. Therefore, we advised the patient to return for assessment every 6 months. However, the patient missed subsequent control appointments and returned one and a half years later.

At the 2-year follow-up, periapical radiography revealed an increase in the size of the periapical radiolucency around the mandibular left central incisor (Figure 2D) compared to that on the previous radiograph. Additionally, the vestibular fistula persisted. Therefore, cone-beam computed tomography (CBCT) was performed to better examine the affected tooth and obtain the correct diagnosis.

CBCT images (Figure 2E) (Planmeca Promax 3d, Helsinki, Finland) showed that teeth #31 and #41 were endodontically treated. No additional root, canal, or foreign periapical material was observed in tooth #31. However, a hypodense heterogeneous lesion with irregular borders was observed. In addition, close examination of the apical area of tooth #31 on a periapical radiograph after adjusting the resolution and brightness revealed radiopaque convexities on the distal aspect of the external root surface associated with the radiolucent area (Figure 2F). Intentional reimplantation (IR) was planned to accurately assess the presence of calculus in the lingual region and rule out the presence of apical cracks or vertical fractures. Informed consent was obtained from the patient prior to the initiation of treatment. All aspects of the procedure, including the rationale, potential risks, benefits, and prognosis, were thoroughly explained to the patient, who then provided written consent for the treatment.

A chlorhexidine mouth rinse was used to control the oral microflora before IR. After achieving complete local anesthesia (Artinibsa 40:10), syndesmotomy of the most coronal fibers of the periodontal ligament was performed using a KAI microscalpel, and the mandibular left central incisor was gently extracted using suitable forceps.

**Figure 3:**
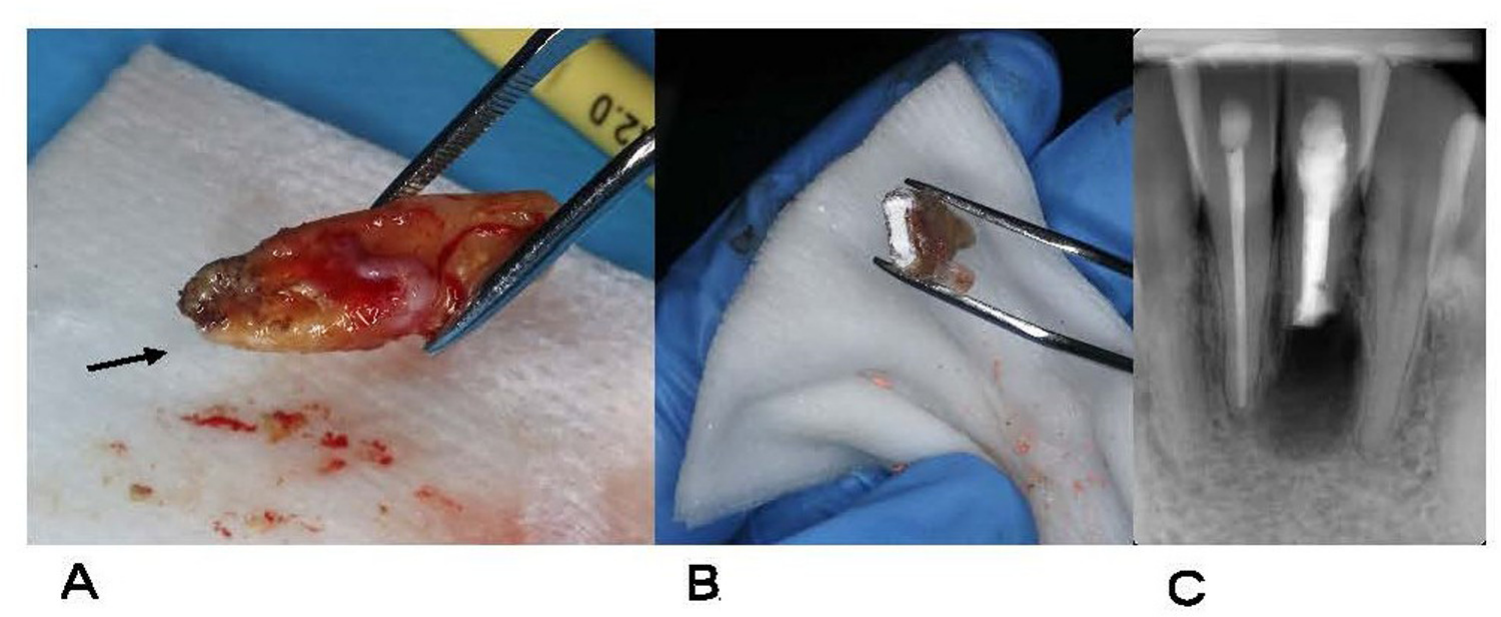
Reimplantation procedure performed on tooth 31. **(A)** After extraction, a dense, dark brown, calculus-like deposit (arrow) firmly attached to the external root surface is observed. **(B)** The calculus-like deposit has been removed, and root-end resection with retrograde preparation and filling of the root apex using bioceramic cement has been performed. **(C)** Postoperative radiograph after reimplantation to verify correct tooth positioning.

The root of the extracted tooth was carefully inspected extraorally. No fissures or fractures were observed on the root surface after staining with methylene blue. However, a dense, dark brown, calculus-like deposit firmly attached to the external root surface was observed (Figure 3A).

After the atraumatic extraction, the calculus-like deposit was removed and the root surface was planed. The tooth was held only by the coronal portion, above the cementoenamel junction, using forceps, while the root surface was wrapped in sterile gauze to avoid direct contact and preserve tissue integrity. Maintaining the tooth hydrated in saline solution at all times, apical resection was performed perpendicular to the long axis of the root using piezosurgical inserts: OT7 for apicoectomy, OT5A for apical planing, and UE1 for cavity preparation (Mectron®, Italy). Curettage was carried out with a Lucas No. 86 curette to remove the granulation tissue. The entire process of root-end resection, retrograde preparation, and retrograde filling was performed under magnification using a Zeiss OPMI Pico microscope (Zeiss, Germany).

Angulation and depth control for the 3 mm retrograde cavity were achieved under high magnification using a surgical microscope. Ultrasonic tips (Proultra, Dentsply Sirona, Switzerland) were used along the long axis of the root canal, and cavity depth was verified using a millimeter-marked periodontal probe and direct visual inspection. This approach ensured proper alignment and standardized preparation prior to retrograde filling. Once the old obturation material was removed, the root surface was re-checked to confirm the absence of cracks or residual extraradicular calculus, and to ensure that no lateral or accessory canals were missed.

For the retrograde filling, a bioceramic material (TotalFill BC RRM Putty, FKG Dentaire, Switzerland) was placed and compacted into the retro-prepared cavity (Figure 3B). This calcium silicate-based cement offers bioactive properties and superior sealing ability. Its putty-like consistency facilitates placement and adaptation. Hu-Friedy pluggers PLGRF1 and PLGRF2 (Hu-Friedy Mfg., Co., LLC, Chicago, IL, USA.) were used for condensation, and the surface of the material was polished using a ball burnisher. Gentle curettage and sterile saline irrigation were performed to clean the extraction socket and sinus tract. The tooth was carefully placed back into its socket, and the buccal and lingual plates were gently pressed. The total extraoral time was 14 min. The fistula was sutured using 5/0 monofilament suture (Supramid; B. Braun, Melsungen, Germany). Subsequently, the patient was instructed to gently bite on a wooden stick to assist tooth stabilization. Correct repositioning of tooth #31 was verified radiographically (Figure 3C), and semi-rigid splints were applied for 2 weeks to support the teeth. Sutures were removed 1 week after the surgical endodontic treatment.

Pharmacological management included amoxicillin 500 mg every 8 h and dexketoprofen 25 mg every 8 h for five days. Additionally, strict oral hygiene was recommended, including rinsing with 0.12% chlorhexidine mouthwash twice daily for 10 days.

**Figure 4:**
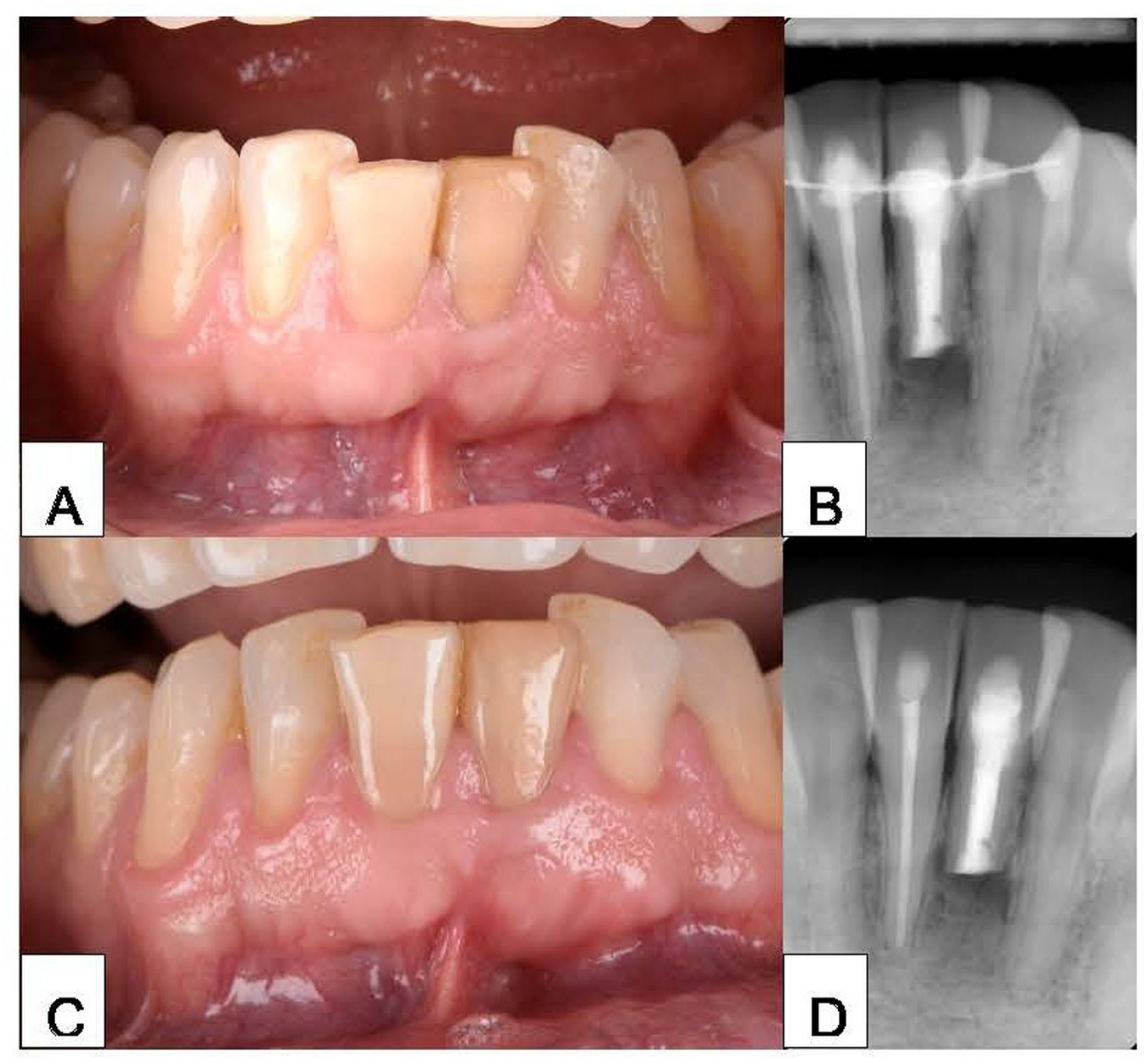
**(A)** Clinical photograph of tooth #31 3 months after IR. **(B)** Periapical radiograph of tooth #31 3 months after IR. **(C)** Clinical photograph of tooth #31 1 year after IR. **(D)** Periapical radiograph of tooth #31 1 year IR. IR, intentional replantation.

At the 3-month recall, resolution of the sinus tract was observed (Figure 4A). Periapical radiography revealed an obvious reduction in the size of the radiolucent area around the treated tooth, with no apical resorption of the root (Figure 4B). On initial radiographic evaluation (CBCT imaging), the periapical lesion associated with the treated tooth presented a well-defined radiolucent area measuring 0.173 cm^3^, consistent with a chronic inflammatory lesion. At 6 months post-replantation, CBCT follow-up revealed a significant reduction in lesion volume to 0.099 cm^3^, along with the appearance of trabecular bone within the cavity, indicative of an active reparative response. At the 1-year follow-up, noticeable improvement was observed clinically (Figure 4C) and radiographically (Figure 4D). At 12 months, radiographic examination showed a minimal residual area of 0.011 cm^3^, with a dense bone pattern and no signs of recurrence, suggesting near-complete and stable osseous healing.

At follow-up examinations, no evidence of external inflammatory or replacement resorption was observed. All extraoral handling was performed in less than 15 min, with care not to touch the remaining root surface, to ensure a favorable prognosis. The patient was asymptomatic with complete healing of the labial mucosa without a sins tract or abscess, and probing depth remained within normal limits at all follow-ups.

## 3 Discussion

Persistent periapical lesions with protracted healing after conventional RCT can be attributed to residual pathogens in the root apex which induce persistent intraradicular infection (17). Furthermore, microorganisms can survive on the external root surface or in the periapical tissue resulting in persistent extraradicular infections (18). In addition, bacterial colonization of root fractures or cracks may lead to extraradicular periapical infections (10). Intra- and/or extra-radicular infections may lead to persistent periapical lesions such as true apical cysts, granulomas, or abscesses (19). Moreover, extrusion of the obturation material or other exogenous substances beyond the apical foramen may cause foreign-body reactions (20, 21), and the local accumulation of endogenous cholesterol crystals can irritate the periapical tissues.

Bacteria develop biofilms on the root surface to protect themselves from environmental factors such as host defense mechanisms, antiseptics, antibiotics, and endodontic cleaning methods (22). Generally, mineralized dental biofilms, which are primarily composed of salts such as calcium phosphate, accumulate between and among the remains of viable microorganisms, leading to the formation of calculus (23). Extraradicular biofilms have been reported in teeth with asymptomatic apical periodontitis, chronic apical abscesses, and sinus tracts (15, 24). Song et al. (11) reported that among teeth with endodontic failure, apical calculus was most frequently observed in the mandibular incisors.

In our patient, an unusual radiopaque lesion was observed in the radiolucent area associated with the apex of the mandibular left central incisor. The findings in this case are consistent with those in a previous study that both foreign-body reactions due to calculus-like deposits and pathogens may cause persistent infections (6). It is important to understand that, with regard to endodontic failure, the 1.8% reported by Song et al. (11) represents the frequency with which the mineralised form of extraradicular biofilm (apical calculus) was surgically identified as a cause of failure in a particular population of previously failed cases, while the estimated 6% reported by Ricucci et al. (10) represents the overall prevalence of extraradicular biofilm in cases of apical periodontitis based on histological studies Despite differences in methodology and the nature of the findings, both studies consistently demonstrate that calcified deposits and extraradicular biofilms are recognized causes of endodontic treatment failure. These factors likely prevented effective resolution of infection with non-surgical endodontic retreatment in our patient.

Upon conducting a visual inspection during the surgical procedure, the deposit was clinically identified as calculiform material, a finding that has been correlated in the literature with persistent apical periodontitis (12, 25). The patient also exhibited a chronic fistulous tract at the time of the initial examination, a prevalent clinical sign associated with persistent periapical infections (10). However, more sophisticated methodologies, such as histological analysis or characterization utilizing techniques like scanning electron microscopy with energy-dispersive x-ray spectroscopy (SEM/EDX) or electron probe microanalysis (EPMA), are requisite for definitive characterization and confirmation of the nature of the deposit. These methodologies facilitate a more precise determination of mineral composition and structure, assisting in the differentiation of true calculus from other mineralized debris (26). In the present case, the exploratory nature of the procedure and/or technical constraints hindered a comprehensive post-extraction analysis of the deposit. Consequently, despite the clinical finding being strongly indicative based on previous reports, the classification of the deposit as mineralized apical calculus remains a clinical diagnosis (12, 25).

A case of unusual radiopacity on the root surface of an endodontically treated maxillary left central incisor associated with a persistent periapical radiolucent lesion has been reported (8). Similar to the findings in our case, pathological investigation revealed calcified particles, such as apical calculus deposits, integrated into the granulomatous tissue (8). The presence of unusual connective tissues with dystrophic calcified areas is the result of an intense body response in an attempt to heal (24).

Previous studies have shown that the sinus tract facilitates communication between the oral cavity and periapical zone, and long-standing fistulas can lead to the formation of apical calculi (16). In such cases, coronal periodontal defects may be absent, and the probing depth may be normal. Extraradicular biofilms are typically an extension of intraradicular infections and are frequently accompanied by observable symptoms such as abscesses or sinus tracts (16). The sinus tract contributes to the delivery of minerals from the saliva to the apical lesion in the absence of periodontal defects (16). This allows free mineral ions and mineral salts to accumulate at the root apex, leading to dental plaque mineralization, periapical inflammation, and formation of unusual calculi (5). The layering of extraradicular deposits may be correlated with the opening and closing of the sinus tract, which results in periods of intense mineralization and decreased activity, respectively (16). This could explain the delayed appearance of apical calcification on radiographs.

The apical deposition of calcium salts is a distinctive and uncommon type of mineralization. However, deposits near the root apex can affect periapical-lesion healing (6). Ricucci et al. (22) emphasized that surgical retreatment should be considered the final option in endodontics to effectively treat periapical lesions. They reported that only surgical retreatment can effectively treat various extraradicular infections associated with symptoms of prolonged exudation that result in endodontic failure. To date, few case reports have described apical periodontitis with a protracted course caused by calculus-like calcium deposits around the root apex. However, in all cases, endodontic retreatment was performed cautiously, and the calculus-like deposit was removed from the root surface of the teeth via periapical flap surgery (6, 8, 24). In our patient, IR, which is another viable treatment option, was performed. IR is particularly advantageous for single-rooted teeth because it involves simple extraction that does not significantly damage the root surface or increase the risk of fracture. IR presents additional advantages, such as the direct clinical observation of inaccessible tooth surfaces (27) and the ability to adequately remove the calculus from those surfaces, which might be hidden during apical surgery, concealing the true reason of failure. However, when handling teeth extraorally, clinicians must take extreme care to avoid drying the root surface (28). IR criteria have evolved over time to include root resection and root-end filling before the tooth is replaced into the socket (29). The tooth's remaining length was roughly 16 mm following apical resection. This was the outcome of a mandibular incisor length of about 20 mm at first, followed by a 3 mm resection and an extra 1 mm lost as a result of the cutting method. As a result, the crown-to-root ratio (CRR) was nearly one to one. It's crucial to remember that, according to the dental literature, a 1:1 CRR is typically accepted as the lowest acceptable level for long-term tooth survival, particularly in particular clinical situations where variables like occlusal load and periodontal health are carefully managed (30). In our patient, radiographic evaluation at the 1-year follow-up revealed satisfactory outcomes (Figure 4D). According to a systematic review and meta-analysis, IR is a viable treatment option with acceptable survival rates for endodontically treated teeth with periapical pathosis (31). The long-term survival rate of teeth with IR ranges from 82.8% to 89.1% (32, 33). The survival of periodontal ligament cells is critical, and reducing extraoral time, ideally to 15 min or less, is associated with higher success rates. The technique employed also influences the outcome.

## 4 Conclusion

The findings in this case suggest that the periradicular tissue fluid or oral fluids infiltrated via the sinus tract promote mineralization of the extraradicular biofilm leading to the formation of calculus-like deposits on the root apex, which contribute to the maintenance of periapical inflammation, serve as a base for the formation of further extraradicular biofilms, and decrease the effectiveness of RCT. Complete removal of the calculus-like deposits via surgical retreatment is required for healing.

## Data Availability

The raw data supporting the conclusions of this article will be made available by the authors, without undue reservation.
